# Cell type-specific changes in Wnt signaling and neuronal differentiation in the developing mouse cortex after prenatal alcohol exposure during neurogenesis

**DOI:** 10.3389/fcell.2022.1011974

**Published:** 2022-12-05

**Authors:** Danielle Sambo, Chiraag Gohel, Qiaoping Yuan, Gauthaman Sukumar, Camille Alba, Clifton L. Dalgard, David Goldman

**Affiliations:** ^1^ Laboratory of Neurogenetics, National Institute on Alcohol Abuse and Alcoholism, National Institutes of Health, Rockville, MD, United States; ^2^ The American Genome Center, Uniformed Services University of the Health Sciences, Bethesda, MD, United States

**Keywords:** prenatal alcohol exposure, single nucleus RNA sequencing, neurogenesis, cortical development, neuronal differentiation, Wnt signaling

## Abstract

Fetal Alcohol Spectrum Disorder (FASD) encompasses an array of effects of prenatal alcohol exposure (PAE), including physical abnormalities and cognitive and behavioral deficits. Disruptions of cortical development have been implicated in multiple PAE studies, with deficits including decreased progenitor proliferation, disrupted neuronal differentiation, aberrant radial migration of pyramidal neurons, and decreased cortical thickness. While several mechanisms of alcohol teratogenicity have been explored, how specific cell types in the brain at different developmental time points may be differentially affected by PAE is still poorly understood. In this study, we used single nucleus RNA sequencing (snRNAseq) to investigate whether moderate PAE from neurulation through peak cortical neurogenesis induces cell type-specific transcriptomic changes in the developing murine brain. Cluster analysis identified 25 neuronal cell types, including subtypes of radial glial cells (RGCs), intermediate progenitor cells (IPCs), projection neurons, and interneurons. Only *Wnt*-expressing cortical hem RGCs showed a significant decrease in the percentage of cells after PAE, with no cell types showing PAE-induced apoptosis as measured by caspase expression. Cell cycle analysis revealed only a subtype of RGCs expressing the downstream Wnt signaling transcription factor *Tcf7l2* had a decreased percentage of cells in the G2/M phase of the cell cycle, suggesting decreased proliferation in this RGC subtype and further implicating disrupted Wnt signaling after PAE at this early developmental timepoint. An increased pseudotime score in IPC and projection neuron cell types indicated that PAE led to increased or premature differentiation of these cells. Biological processes affected by PAE included the upregulation of pathways related to synaptic activity and neuronal differentiation and downregulation of pathways related to chromosome structure and the cell cycle. Several cell types showed a decrease in Wnt signaling pathways, with several genes related to Wnt signaling altered by PAE in multiple cell types. As Wnt has been shown to promote proliferation and inhibit differentiation at earlier stages in development, the downregulation of Wnt signaling may have resulted in premature neuronal maturation of projection neurons and their intermediate progenitors. Overall, these findings provide further insight into the cell type-specific effects of PAE during early corticogenesis.

## 1 Introduction

Prenatal alcohol exposure (PAE) is the leading preventable cause of neurodevelopmental disability in the western world (Almeida et al., 2020). Despite public health efforts to discourage alcohol use during pregnancy, there has been an increase in both current drinking (from 9.2% to 11.3%) as well as binge drinking (from 2.5% to 3.9%) among pregnant women in the United States in recent years (Denny et al., 2020). The negative consequences of PAE include neurological, cognitive, physical, and behavioral deficits, collectively identified under the umbrella term fetal alcohol spectrum disorder (FASD). It is estimated that 10 in every 1,000 children born in the United States manifest FASD (May et al., 2018). Heavy alcohol use during pregnancy can result in fetal alcohol syndrome (FAS), the most severe form of FASD, which entails the presentation of growth restriction, facial dysmorphology, and central nervous system structural and functional abnormalities (Wozniak et al., 2019). Moderate drinking is estimated to affect 5%–20% of births, and although not typically leading to the physical hallmarks of FAS, it can induce alcohol-related neurological disorders (ARND) (Olguin et al., 2021). ARND symptoms persist into adulthood and include impairments in cognitive abilities, social skills, and adaptive functioning (Wozniak et al., 2019).

Dysregulation of cortical development is thought to be a major contributor to FASD because many of the social, affective, and cognitive deficits associated with PAE are regulated by cortical regions (Fischer et al., 2021). The cortex is particularly vulnerable to the effects of PAE (Mathews et al., 2021), and essentially all cellular processes that occur during neurogenesis, including proliferation, differentiation, and migration, have been shown to be affected by PAE in preclinical studies (Almeida et al., 2020). The type and severity of neurodevelopmental disturbances resulting from PAE is dependent on the timing, dose, and duration of the teratogenic insult. PAE during the first trimester is primarily associated with facial and structural abnormalities (Sulik et al., 1981), while PAE during the third trimester is associated with increased vulnerability of neurons to neurodegeneration (Saito et al., 2016). PAE during cortical neurogenesis, which spans the mid-first trimester to second trimester in humans (Workman et al., 2013), is associated with abnormal distribution of cortical layers and cortical thinning (Miller, 1993; Camarillo and Miranda, 2008). Brain imaging in individuals subject to PAE have revealed lasting disturbances in brain growth long after the prenatal exposure (Riley et al., 2004).

Several diverse mechanisms by which ethanol (EtOH) elicits its harmful effects in the brain have been explored, including nutritional deficiencies, epigenetic alterations, disruption of cell-cell interactions and decreased cell adhesion molecules, dysregulated growth factor and morphogen signaling, and oxidative stress leading to apoptosis (Bhatia et al., 2019). Given the wide spectrum of phenotypic outcomes of PAE, multiple molecular mechanisms working in concert likely contribute to FASD. While the effects of heavy PAE have been widely explored, the effects of more moderate exposures are less understood. To explore the teratogenic effects induced by moderate PAE during early cortical development, we used single nucleus RNA sequencing (snRNAseq) to measure the cell type-specific effects of PAE on gene expression during the onset of mouse neurulation (embryonic day 8, E8) midway through cortical neurogenesis (E14). Single cell RNAseq (scRNAseq) and snRNAseq allow for the unbiased survey of transcriptomic profiles of individual cells and is thus a powerful tool for revealing cell type-specific cellular changes within a heterogenous tissue such as the brain. Importantly, such an approach may reveal teratogenic effects of moderate PAE that would otherwise be masked by averaging the effects across diverse cell types or selectively focusing on specific cell types. Overall, we found cell type-specific vulnerabilities in response to moderate PAE that implicate a downregulation of Wnt signaling and augmentation of neuronal differentiation, suggesting potential mechanisms by which cortical development is dysregulated in FASD.

## 2 Materials and methods

### 2.1 Animal use and prenatal alcohol exposure

All procedures used in this study were approved by the Institutional Animal Care and Use Committee at the National Institute on Alcohol Abuse and Alcoholism Intramural Research Program. C57BL/6J mice (Jackson Laboratory) were maintained at a 12 h: 12 h light: dark cycle and given access to food and water *ad libitum*. Timed matings were set up in the evening between a single male and single female. Females were separated the following morning (embryonic day 0.5 or E0.5) and checked for a copulation plug. Female weight was monitored, and pregnant dams were used for prenatal alcohol or saline exposure. On E8.5, pregnant females were treated with either saline or 2.5 g/kg EtOH, delivered in 25% v/v 200 proof ethanol in saline, by intraperitoneal injection (i.p.) once daily for 6 days (E8.5 to E13.5). The dose of EtOH chosen is estimated to result in a blood ethanol concentration (BEC) of 150 mg/dl–200 mg/dl (Schambra et al., 2017) and is considered a moderate to high dose of EtOH. On E14.5, dams were euthanized by cervical dislocation, and embryos were collected for dissection. Cerebral cortices from both halves were isolated, flash frozen, and stored at −80°C. Six cortices were selected at random from 2 separates dams per condition for single nucleus RNA sequencing (snRNAseq).

### 2.2 Nuclei preparation

Nuclei were isolated from frozen tissue using a previously validated protocol (Bakken et al., 2018). Briefly, frozen tissue was homogenized in a homogenization buffer containing 10 mM Tris pH 8.0, 250 mM sucrose, 25 mM MgCl_2_, 0.1% Triton-X 100, 0.5% RNasin Plus RNase Inhibitor (Promega, N2611), 1X Protease Inhibitors (Promega G6521), and 0.1 mM DTT using a motorized pestle homogenizer. Homogenates were strained through a 30 μM cell strainer (Falcon, 352,235) followed by centrifugation at 300 rcf for 10 min to pellet nuclei. Pellets were then washed twice in buffer containing 1X PBS without Mg or Cl_2_, 0.4% UltraPure nuclease-free BSA (Life Technologies, AM2616), and 0.5% RNasin Plus. Final nuclei suspensions were counted using the Countess II (Thermo Fisher).

### 2.3 Single-nucleus RNA-sequencing library preparation and sequencing

10,000 nuclei were loaded on to the Chromium Controller (10X Genomics) for generation of single nuclei barcoded droplets using the Single Cell 3′ V3.1 Reagent Kit. Libraries were constructed from droplets using the 10X Single Cell Version 3.1 chemistry per the manufacturer’s instructions. Sequencing libraries were quality assessed for size distribution and adapter artifacts using the Bioanalyzer (Agilent) and Fragment Analyzer (Agilent) and quantified using a KAPA Library Quantification Kit (Roche, KK4873). Sequencing libraries were prepared across two different batches. Libraries were normalized and pooled before quantification of the pool by real-time PCR and a KAPA Library Quantification Kit (Roche, KK4854) and sequenced on the NovaSeq 6,000 Sequencing System (Illumina) using a 200-cycle kit. 67,000–83,000 reads per nuclei were obtained.

### 2.4 Data analysis

#### 2.4.1 Data processing

The 10X Genomics Cell Ranger Single Cell pipeline was used to demultiplex samples, process and align barcodes, and integrate samples using the default and recommended parameters. Cellranger’s default mm 10 reference dataset was used for alignment. Seurat (4.1.0) was used for quality control and downstream analysis. Cells were filtered out based on having the following criteria: 1) less than 500 or greater than 10,000 genes; 2) greater than 2.5% mitochondrial genes; 3) greater than 50% ribosomal genes; 4) greater than 0.075% hemoglobin genes; and 5) greater than 100,000 RNA count. Genes were filtered out that: 1) were expressed in less than 10 cells; 2) known to produce bias including sex-specific genes (*Gm42418, AY036118, Gm47283, Rpl26, Gstp1, Rpl35a, Erh, Slc25a5, Pgk1, Eno1, Tubb2a, Emc4, Scg5, Ehd2, Espl1, Jarid1d, Pnpla4, Rps4y1, Xist, Tsix, Eif2s3y, Ddx3y, Uty, Kdm5d, Cmss1, AY036118*, and *Gm47283*) (Romanov et al., 2020); and 3) mitochondrial genes, which are not expected to be expressed in nuclei (*mt-Nd2, mt-Atp6, mt*-*Co1, mt-Co2, mt-Co3, mt-Cytb, mt-Nd1, mt-Nd4, mt-Nd2,* and *mt-Nd2*). Data was normalized using SCTransform, and dimensionality reduction was performed using principal component analysis (PCA). Harmony was used to correct for the effect of batch and mouse (dam).

#### 2.4.2 Unbiased clustering and cell type identification

Nearest neighbor graphs were generated using 30 PCs after Harmony correction, and clusters were found using the smart local moving (SLM) algorithm with a resolution of 0.8. One saline sample was removed for consistently having a significantly different distribution of cells in the identified clusters compared to all other samples. Clusters were numbered in order of decreasing cell count. FindMarkers (Seurat) was used to identify marker genes for each cluster. Marker genes with a *p*-value < 0.05 were sorted by decreasing log fold change to identify each cluster. Clusters were classified into cell types by comparing marker genes with other scRNAseq cell type marker data from E14.5 mouse cortex (Loo et al., 2019; Bedogni and Hevner, 2021; Ruan et al., 2021) (Supplemental Material S1). To compare the percentage of cells in each cluster for saline and EtOH treated embryos, clusters were separated into 5 groups of 5 clusters by cell count, and two-way ANOVA with Bonferroni’s multiple comparison was used to identify statistical differences.

#### 2.4.3 Cell cycle analysis

CellCycleScoring from the Seurat package was used to assign cells into the G2/M, S, or G1 phase of the cell cycle based on gene expression data (Supplemental Material S2). The percentage of cells in each cell cycle phase for each cluster and sample was quantified, and comparison of saline vs. EtOH treatment was performed for each phase using two-way ANOVA with Bonferroni’s multiple comparison.

#### 2.4.4 Pseudotime analysis

The R package Monocle3 was used for pseudotime analysis. The dataset was separated by treatment. SCTransform normalization, PCA, and Harmony correction on the “batch” variable were applied to each subset. Separate UMAPs were generated from the saline samples and EtOH samples to derive pseudotime scores. “Get_earliest_principal_node”, a helper function provided by Monocle3, was used to identify a principal node associated with Cluster 21 as the starting point for cell trajectory graphs for both saline and EtOH. Bootstrap difference in pseudotime means between saline and EtOH was performed as follows: 1) Sample n pseudotime scores from all ethanol cells with replacement, where n is the number of EtOH cells, and calculate the mean of these sample scores. 2) Sample m pseudotime scores from all saline cells with replacement, where m is the number of saline cells, and calculate the mean of these sample scores. 3) Find the differences between these two mean values. Steps 1–3 were repeated 10,000 times to achieve a difference in means distribution. Comparison of saline vs. EtOH treatment average pseudotime scores was performed using two-way ANOVA with Bonferroni’s multiple comparison. Wasserstein distance between saline and EtOH pseudotime scores was calculated using the transport package from CRAN.

#### 2.4.5 Differential gene expression analysis

Analysis of differentially expressed genes (DEGs) between saline and EtOH treatments was performed for each cluster using the R package DESingle. DEGs with a fold-change greater than 1.25 or less than 0.8, and an adjusted *p*-value < 0.05 were considered significant. DEtype subdivides the DEGs into 3 types: DEs, DEa and DEg. DEs refers to genes that show a significant difference in the proportion of real zeros in the two groups, but do not have a significant difference in the other cells. DEa refers to genes that are significantly differentially expressed between the groups without significant difference in the proportion of real zeros. DEg refers to genes that have a significant difference in both the proportions of real zeros and the expression abundances between the two groups. All DE gene types were used when reporting DE results.

#### 2.4.6 Pathway analyses

To determine pathways implicated by the DEG analysis, Gene ontology (GO) enrichment analysis was performed using goana from the limma package. Kyoto Encyclopedia of Genes and Genomes (KEGG) pathway enrichment analysis was performed using kegga from the limma package. GO terms were considered significantly different that had a minimum of 3 genes present and *p*-value < 0.01. KEGG pathways were considered significant that had a minimum of 3 genes.

#### 2.4.7 Gene expression

Bootstrap analysis was performed by resampling the normalized feature x cell vector for a given gene with replacement and calculating the mean and standard deviation of the resamples 10,000 times. Caspase count analysis was performed by calculating the proportions of cells in a given sample and cluster that have a Casp3 transcript count greater than n, where *n* ∈ (1,2,3,4,5).

## 3 Results

### 3.1 Cell types identified by single nucleus RNA sequencing in the developing cortex

**FIGURE 1:**
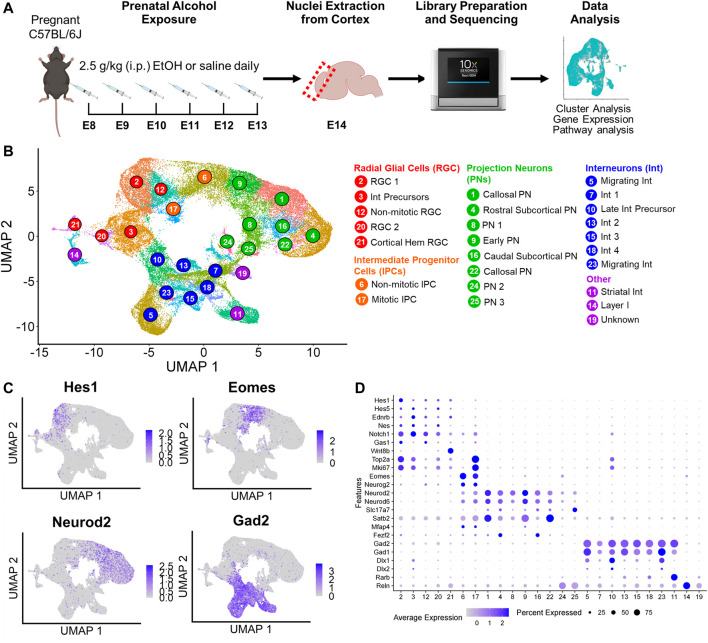
Experimental design and cluster identification. **(A)** Schematic of the prenatal alcohol exposure (PAE) paradigm. The icons used were obtained from BioRender.com. **(B)** Uniform Manifold Approximation and Projection (UMAP) visualization of the 25 clusters identified. **(C)** UMAPs overlaid with gene expression of canonical cell type markers (*Hes1*: radial glial cells; *Eomes*: intermediate progenitor cells; *Neurod2*: projection neurons; *Gad2*: interneurons). **(D)** Dot plot representation of the average expression and percentage of cells expressing markers of cluster cell types. Darker dots indicate higher gene expression, and larger dot sizes indicates higher percentage of cells expressing the gene.

To investigate the effects of PAE on cell type-specific changes in the developing cortex, pregnant C57BL/6J mice were exposed to saline or 2.5 g/kg (i.p.) EtOH daily for six days from embryonic day 8 (E8) to E13, corresponding with the onset of neurulation through cortical neurogenesis (Martynoga et al., 2012). On E14, cortices were extracted from embryos and nuclei were isolated for snRNAseq (Figure 1A). A total of 53,990 nuclei were analyzed, with an average of 8,318 transcripts per nucleus and 2,847 genes per nucleus included in downstream analysis after quality control (Supplementary Table S1). Sequencing metrics were on par with similar scRNAseq studies (Loo et al., 2019).

Initial cluster analysis identified 33 principal cell types ranked by abundance (Supplementary Figure S1A). Clusters were identified using differential gene expression analysis and annotated based on canonical cell type markers and previously published transcriptomic data from the E14 murine cortex (Loo et al., 2019; Bedogni and Hevner, 2021; Ruan et al., 2021). Of the 33 clusters, Cluster 15 was excluded due to low transcript count (Supplementary Figure S1B). Cluster 22 was identified as *Ntng1*+/*Tcf7l2*+ thalamic cells that showed biased expression in only three of the samples (Supplementary Figure S1C), likely due to variability in the collection of tissue adjacent to the cortex; therefore, Cluster 22 cells were also excluded. Five of the 33 clusters were non-neuronal cell types, including Cluster 27 *Col1a1*+/*Cxcl12*+ meningeal fibroblasts, Cluster 30 *Mcam*+/*Rgs5*+ pericytes, Cluster 31 *P2ry12*+/*Ly86*+ microglia, Cluster 32 *Cd93*+/*Pecam*+ endothelial cells, and Cluster 33 *Pdgfra*+/*Olig2*+ oligodendrocyte precursor cells (OPCs) (Supplementary Figure S1D). These clusters, as can be inferred by their low rank abundances, represented a small percentage of the total cells (1.3% meningeal fibroblasts, 0.34% pericytes, 0.24% microglia, 0.10% endothelial cells, and 0.08% OPCs) with no differences in cell percentages between saline and EtOH detected in these clusters. Furthermore, the low cell count in these clusters decreased the power to detect statistically significant differentially expressed genes (DEGs), and DEG analysis comparing saline and EtOH in these clusters failed to identify any genes. Therefore, the non-neuronal cells were removed from further analyses.

A second cluster analysis was performed on the remaining neuronal and neuronal progenitor cell types, and 25 principal cell types were identified (Figure 1B). These clusters were further categorized into major cell types: *Hes1*+ radial glial cells (RGCs) (5 clusters), *Eomes* + intermediate progenitors (IPCs) (2 clusters), *Neurod2*+ projection neurons (PN) (8 clusters), and *Gad2*+ expressing interneurons (Int) (7 clusters). Expression of these major cell type markers overlaid on Uniform Manifold Approximation and Projections (UMAPs) revealed separation of the identified clusters into these broad cell type categories (Figure 1C). In addition to these major cell types, *Rarb*+ striatal interneurons (Cluster 11) and *Reln*+ Layer I Cajal-Retzius cells (Cluster 14) were also identified. While Cluster 19 expressed pan-neuronal markers including *Dcx* and *Map2* (Supplementary Figure S2), this cluster did not express *Neurod2* or *Gad2* and therefore was not classified as projection neurons or interneurons and was considered an unknown neuronal cell type for this study.

### 3.2 Characterization of cluster cell types

Gene expression analysis enabled further classification of some of the clusters (Figure 1D; Supplementary Figure S2). Proliferation markers *Top2a* and *Mki67* were highly expressed in most RGC clusters as well as one IPC (Cluster 17) and one interneuron cluster (Cluster 10, described further below). Based on expression of these markers, IPC Cluster 17 was identified as mitotic IPCs and IPC Cluster 6 as non-mitotic. RGC Cluster 12 displayed low expression of proliferation markers and was classified as non-mitotic RGCs. RGC Cluster 3 expressed both RGC markers as well as interneuron transcription factors *Dlx1* and *Dlx2*, suggesting this cluster may represent interneuron precursors. RGC Cluster 21 revealed elevated expression of cortical hem markers *Wnt8b* and *Rspo2* as well as RGC markers *Hes1*, *Hes5*, *Ednrb*, and *Nestin*, therefore this cluster was classified as cortical hem RGCs.

Neuronal clusters expressed pan-neuronal markers *Dcx* and *Map2*. Of the projection neurons, *Satb2*, a marker for long-range callosal projection neurons (Arlotta et al., 2005), was highly expressed in Clusters 1 and 22, with intermediate expression in Cluster 9. Cluster 9 also had moderate expression of the intermediate progenitor marker *Mfap4*, suggesting Cluster 9 may represent early projection neurons. Clusters 4 and 16 expressed higher levels for *Fezf2*, a marker for subcortical projection neurons (Chen et al., 2008), with Cluster 4 expressing the rostral marker *Mc4r* and Cluster 16 expressing the caudal marker *Crym* (Loo et al., 2019). Of the seven interneuron clusters, Cluster 5 and 23 also expressed high levels of *Erbb4*, a putative marker for migrating interneurons (Yau et al., 2003). Cluster 10 expressed proliferation markers *Top2a* and *Mki67* as well as interneuron precursor markers *Dlx1* and *Dlx2.* Compared to interneuron precursor Cluster 3, Cluster 10 expressed *Gad2.* Therefore, this cluster likely represents later stage interneuron precursors and was therefore grouped with the other interneuron clusters. The remaining clusters not further classified were identified by their major cell type (i.e., Cluster 2 as “RGC 1”).

### 3.3 PAE decreases *Wnt*-expressing progenitor cells

**FIGURE 2:**
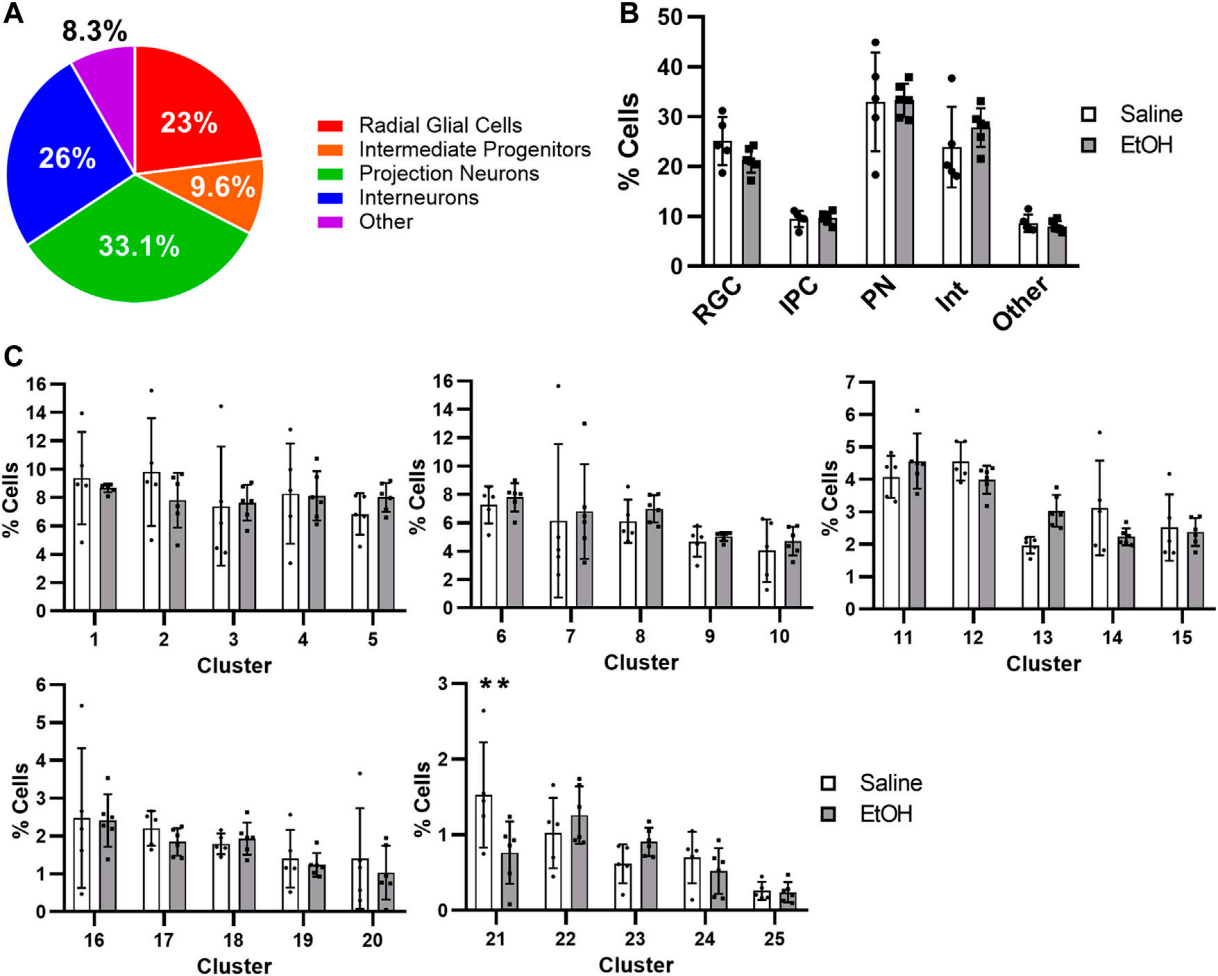
Distribution of cell across cell types. **(A)** Pie chart of the percentage of major cell types across all samples. **(B)** Bar graph of the percentage of cells in each major cell type comparing saline and EtOH. **(C)** Bar graphs of the percentage of cells for each cluster comparing saline and EtOH. The 25 clusters were grouped in cohorts of 5. ANOVA **: *p*-value < 0.01.

The distribution of PAE-exposed and non-exposed cortical cells across major cell types was similar to previous reports from murine E14 cortices (Figure 2A) (Loo et al., 2019), with the largest proportions of cells constituting PN (33.1%), followed by progenitors (23% RGCs and 9.6% IPCs totaling 32.6%), interneurons (26%), and other cell types (8.3%). We found no change in the percentages of major cell types after PAE (Figure 2B), however cortical hem RGC Cluster 21 were significantly decreased by roughly 40% after EtOH treatment (Figure 2C). No other cluster had significantly altered percentages of cells; thus, cortical hem RGCs may represent a cell type vulnerable to the effects of moderate PAE in the treatment paradigm used. Cluster 21 cortical hem RGCs showed marked expression of *Wnt* genes (Supplementary Figure S3), and the effects of PAE on Wnt signaling have also been observed in previous FASD studies (Hashimoto-Torii et al., 2011; Muralidharan et al., 2018; Chater-Diehl et al., 2019; Fischer et al., 2021).

To determine whether apoptosis was induced in any cell type, we measured expression of caspase-3 (*Casp3*), the primary caspase implicated in PAE-induced apoptosis (Dong et al., 2010). Normalized expression of *Casp3* revealed no change in expression after PAE (Supplementary Figure S4A). Furthermore, when determining the percentage of cells expressing *Casp3* transcripts, on average, in 75% of the cells from either treatment group expression of the transcript was undetectable and only 5% expressed more than 2 copies (Supplementary Figure S4B). This finding is consistent with a previous scRNAseq study also reporting no significant apoptosis as measured by caspase expression (Salem et al., 2021) as well as reports that higher doses of EtOH may be required for measurable apoptosis in rodent models (Almeida et al., 2020). Of note, because dying cells are likely excluded from sn- and scRNAseq due to low quality reads or transcript counts, these technologies may not accurately reflect the extent of apoptosis. Although the percentage of cells in Cluster 21 cortical hem RGCs was decreased after PAE (Figure 2C) and Cluster 21 did not show elevated *Casp3* expression, the decrease in this cell type may have taken place at time points prior to tissue collection or single nucleus preparation and in that case would be no longer detectable.

### 3.4 PAE alters the cell cycle progression of *Tcf7l2*-expressing radial glial cells

**FIGURE 3:**
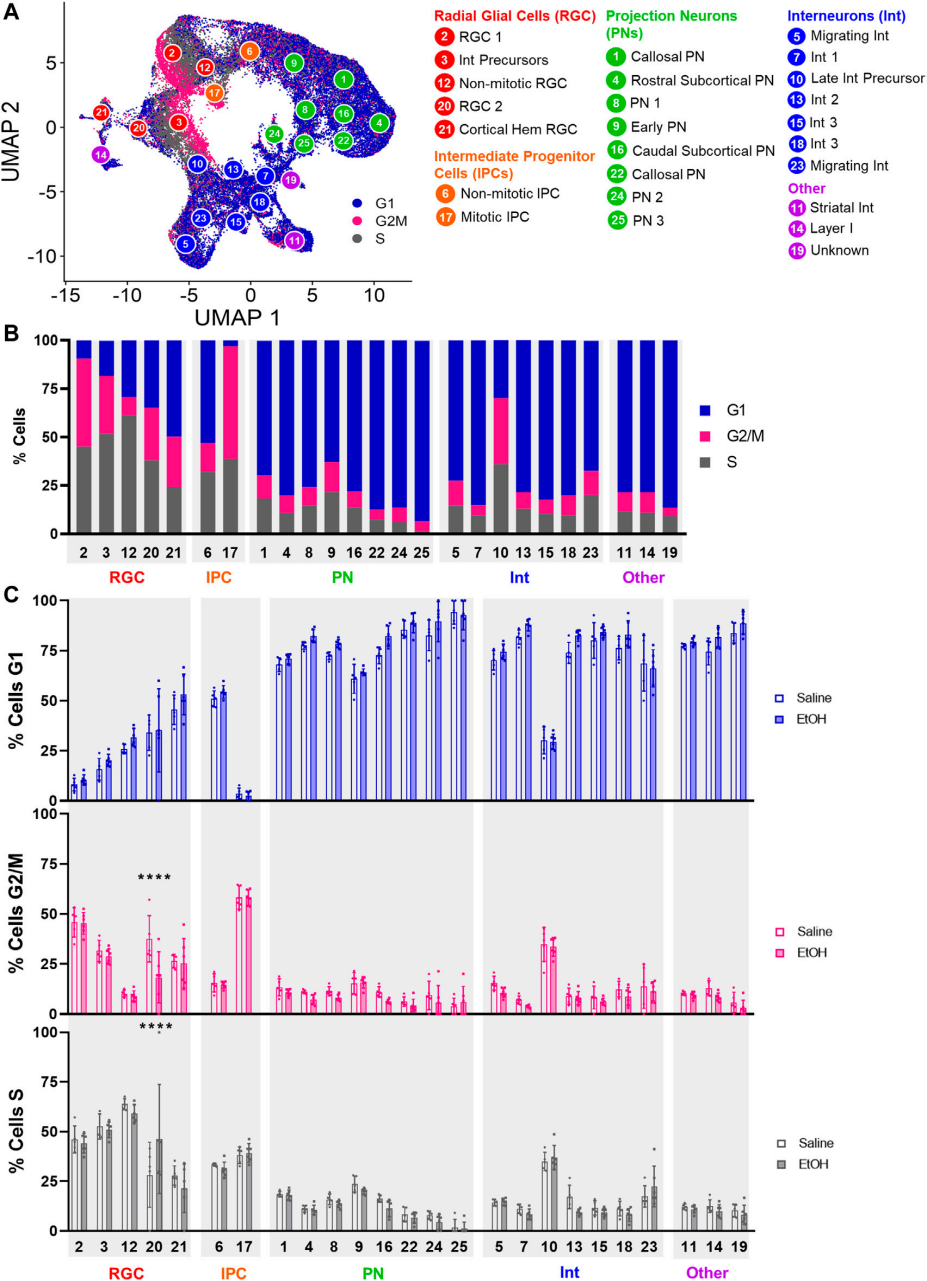
Percentage of cells in cell cycle stages. **(A)** UMAP overlaid with the cell cycle stage for each cell. **(B)** Bar graph of the percentage of cells in each cell cycle stage across all samples. **(C)** Bar graph of the percentage of cells in each cell cycle stage comparing Saline and EtOH. ****: *p* < 0.0001.

We then investigated whether PAE altered the distribution of cells within different phases of the cell cycle. Cells were assigned to the G1, G2/M, or S phase of the cell cycle using ccSeurat. As shown in Figures 3A,B, progenitor cell types displayed the highest percentage of cells in the G2/M phase, consistent with the proliferative capacity of these cells, whereas the projection neuron and interneuron clusters were predominately in G1. Comparison of saline and EtOH treatment groups revealed a significant difference in the distribution of cells in the cell cycle for RGC Cluster 20 (Figure 3C), which showed a decrease in the percentage of cells in G2/M and increased percentage of cells in the S phase of the cell cycle, suggesting impaired proliferation of this RGC cell type. Cluster 20 cells robustly expressed *Tcf7l2*, a transcription factor downstream in the Wnt signaling pathway, relative to the other clusters as well as other Wnt pathway transcription factors (Supplementary Figure S3). The decreased percentage of Cluster 21 *Wnt*-expressing cells and decreased percentage of mitotic cells in Cluster 20 *Tcf7l2*-expressing cells is consistent with the reported role of Wnt signaling in increasing the expression of genes that promote proliferation (Chenn, 2008) and further implicates Wnt signaling in the effects of PAE, particularly on progenitor cell types.

### 3.5 PAE increases differentiation in a cell type-specific manner

**FIGURE 4:**
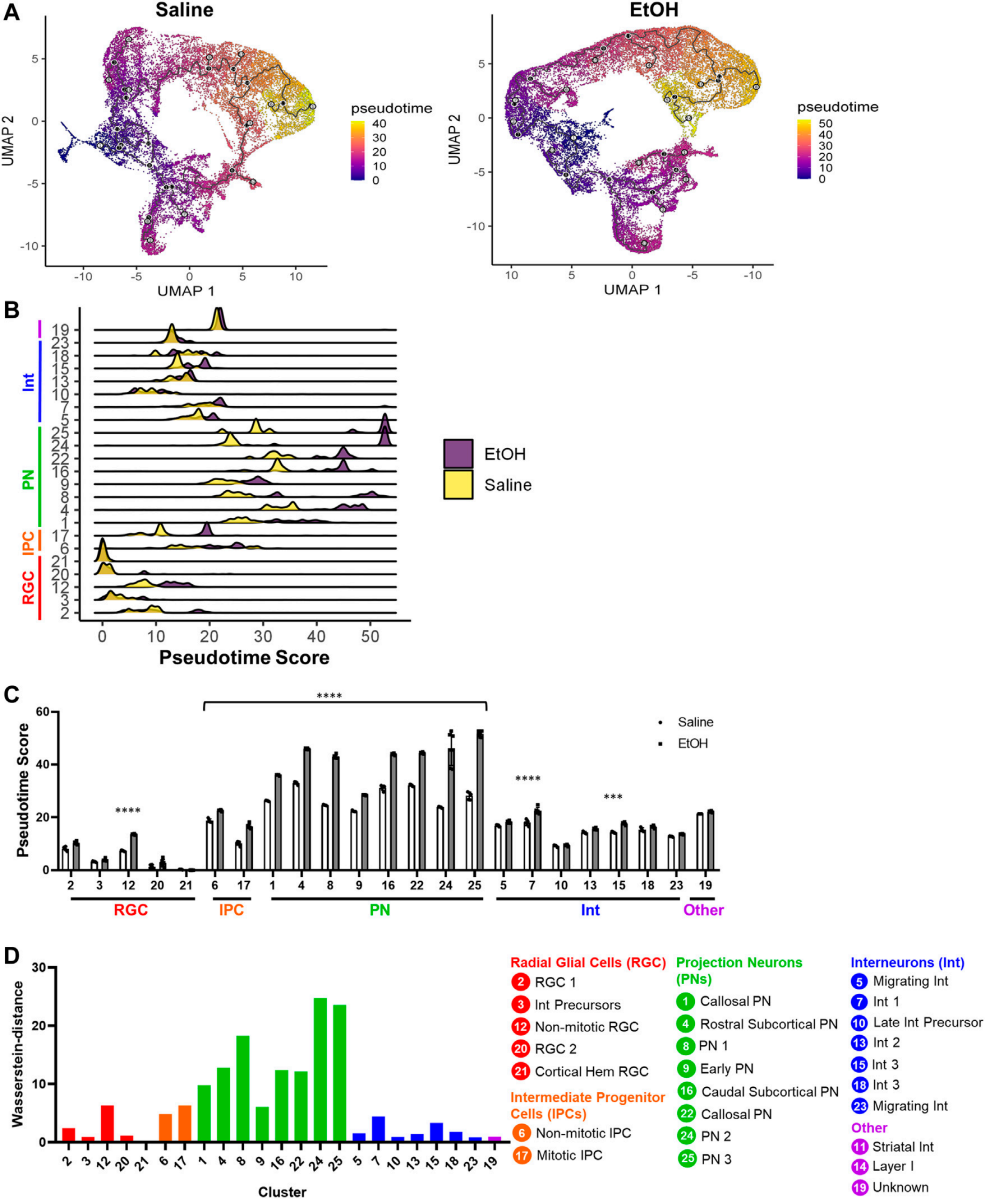
Pseudotime analysis of cell differentiation trajectories. **(A)** Individual UMAPs for Saline and EtOH overlaid with color representative of pseudotime scores for each cell. **(B)** Bridge plot of the pseudotime score distribution for cells within each cluster. **(C)** Bar graph of the average pseudotime score for each cell type comparing Saline and EtOH. **(D)** Wasserstein-distance between the distribution of Saline and EtOH pseudotime scores for each cluster. ***: *p* < 0.001. ****: *p* < 0.0001.

We next investigated the effect of PAE on differentiation progression using pseudotime analysis. In order to construct trajectory graphs to determine pseudotime scores for each treatment, separate UMAPs were generated for saline and EtOH samples (Figure 4A). *Rarb*+ striatal interneurons (Cluster 11) and Layer I *Reln*+ Cajal-Retzius cells (Cluster 14) were removed for this analysis since these are outlying cell types of distinct lineage (Bielle et al., 2005; Lim et al., 2018). As expected, progenitor cell clusters showed lower pseudotime scores compared to neuronal clusters, with PNs showing the highest pseudotime scores. Furthermore, the expression of genes known to be related to neuronal maturation were increased in clusters with increasing pseudotime scores (Supplementary Figure S5). When comparing saline and EtOH, we found a significant increase in the average pseudotime scores in all IPC and PN clusters as well as the non-mitotic RGC Cluster 12 and interneuron clusters 7 and 15 (Figures 4B,C). Because the pseudotime scores across cells in each cluster does not follow a normal distribution, we also measured the Wasserstein distance between the treatment groups, which is a measure of the minimum movement required to transform the shape of one probabilistic distribution to another (Gan et al., 2022). The Wasserstein distances measured were consistent with the average pseudotime score findings, where the IPCs, PNs, and select RGC and interneuron clusters showed a greater Wasserstein difference compared to other clusters (Figure 4D). These results suggest PAE pushed IPCs and PNs as well as non-mitotic RGCs and specific subtypes of interneurons further along in their developmental trajectories. The premature maturation of glutamatergic neurons is consistent with previous reports in other FASD models, as detected by the upregulation of markers for neuronal maturation (Kim et al., 2010; Gil-Mohapel et al., 2011; Zhu et al., 2017). Of the affected RGC cell types, only the non-mitotic cluster showed increased differentiation, suggesting cell state-specific effects of PAE on differentiation.

### 3.6 Cell type-specific gene expression alterations after PAE

**FIGURE 5:**
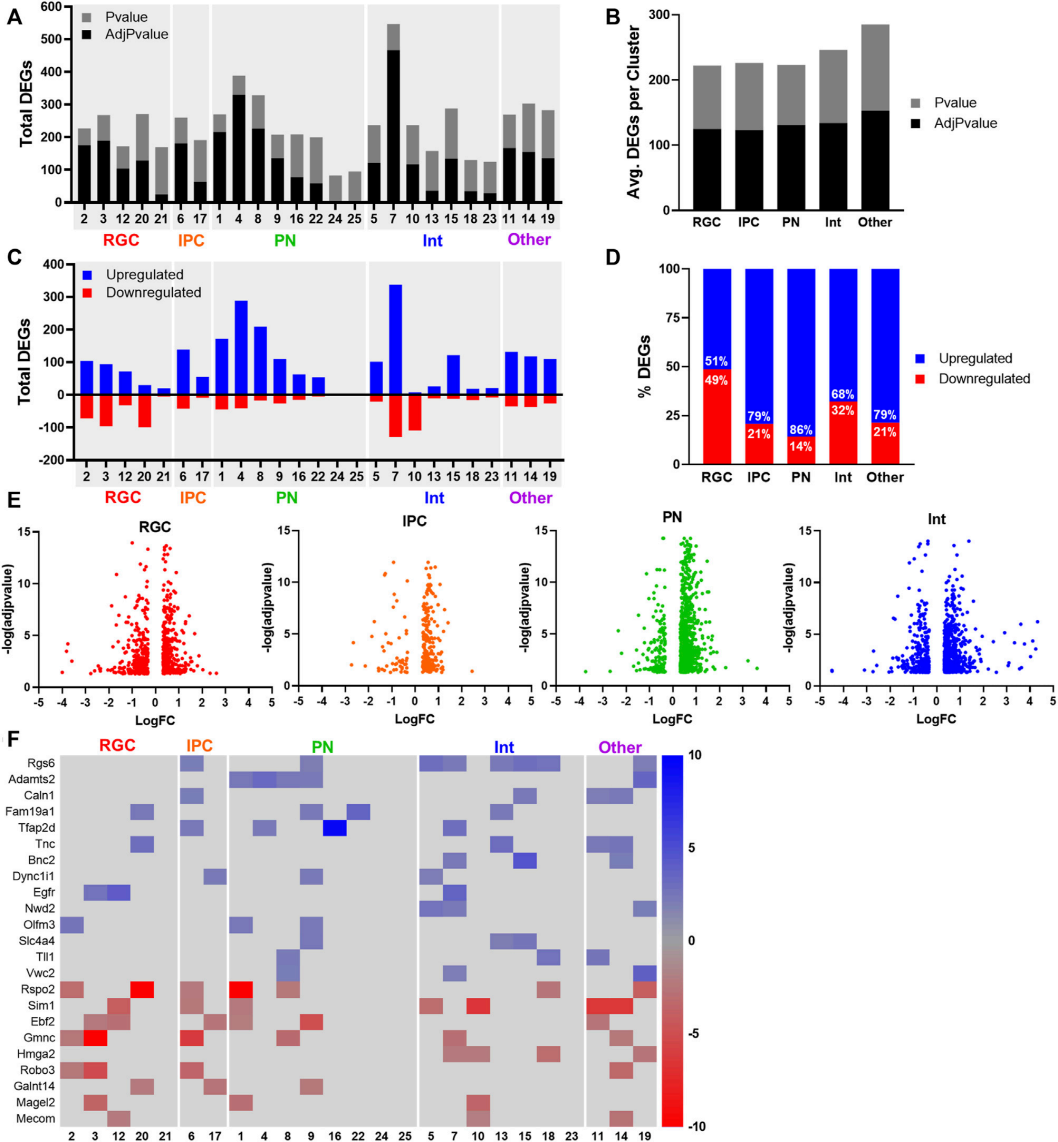
Differentially expressed genes after PAE. **(A)** Total nominal *p*-value (*p* < 0.05) and adjusted *p*-value (adj.*p* < 0.05) significant differentially expressed genes (DEGs) per cluster. **(B)** Average number of nominal *p*-value (*p* < 0.05) and adjusted *p*-value (adj.*p* < 0.05) significant DEGs per cluster for major cell types. **(C)** Total downregulated (red) and upregulated (blue) DEGs per cluster. **(D)** The percent of upregulated and downregulated DEGs per major cell type. **(E)** Volcano plots of the log fold change (logFC) and–log of the adjusted pvalue [-log (adjpvalue)] for all DEGs in major cell types. **(F)** Heatmap the fold change of DEGs signficant in at least 3 clusters with fold change > 2.

Differential gene expression analysis was performed to identify genes and pathways altered by PAE (Figure 5A). Of the major cell types, the average number of differentially expressed genes (DEGs) per cluster was similar for RGCs, IPCs, PNs, Int, and the other cell types (Figure 5B). We found an overall increased expression of DEGs after EtOH treatment compared to saline (Figure 5C), with 907 total downregulated DEGs and 2,410 total upregulated DEGs. This trend towards upregulated DEGs after PAE is consistent with previous studies (Zhu et al., 2017; Fischer et al., 2021). Interestingly, when combining clusters within major cell types, the bias towards gene upregulation was most observed in the PN, followed by IPCs and interneurons, whereas RGCs displayed a more equal distribution of up- and downregulated DEGs (Figures 5D,E). The cluster with the highest number of DEGs (547) and highest number of upregulated DEGs (392) was Interneuron Cluster 7, which was also one of the two interneuron clusters that showed increased pseudotime scores with PAE. The top marker gene for Cluster 7 was *Snhg11*, a long non-coding RNA (lncRNA) primarily implicated in cancer cell progression. Interestingly, Snhg11 has been shown to promote cancer progression *via* activation of Wnt signaling (Liu et al., 2020; Wu et al., 2021). Previous scRNAseq studies in E14 cortex have classified *Snhg11* as a novel interneuron marker (Loo et al., 2019), however its function in interneurons or brain development is largely unknown.

Although the majority of DEGs were unique to the clusters which they were significant, some genes were significant across multiple cell types. Of the upregulated repeating DEGs with the highest fold change (Figure 5F) were genes implicated in neuronal differentiation and neurogenesis (*Rgs6*, *Fam19a*, *Olfm3*, and *Vwc2*) and neurite outgrowth and axon development (*Tnc*, *Dync1i1*). *Egfr* (epidermal growth factor receptor), a gene widely associated with cell migration, adhesion, and proliferation, was upregulated in RGC clusters 3 and 12 and Interneuron cluster 7 and has been previously implicated in the effects of PAE in multiple tissues including the brain (Green et al., 2007). Genes recurrently downregulated across cell types with the highest fold change included several involved in the regulation of the cell cycle (*Gmnc*, *Hgma*, and *Magel2*), midline axon guidance (*Robo3*), and Wnt signaling (*Rspo2*, *Ebf2*).

### 3.7 Biological processes altered by PAE

**FIGURE 6:**
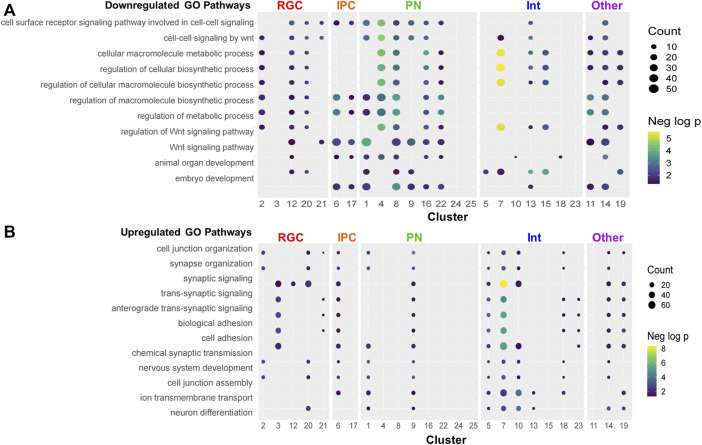
Gene ontology analysis of pathways altered by PAE. **(A–B)** Dot plot of the top repeating downregulated **(A)** and upregulated **(B)** pathways. Pathways were considered significant with a *p*-value < 0.05 and containing at least 3 DEGs.

**FIGURE 7:**
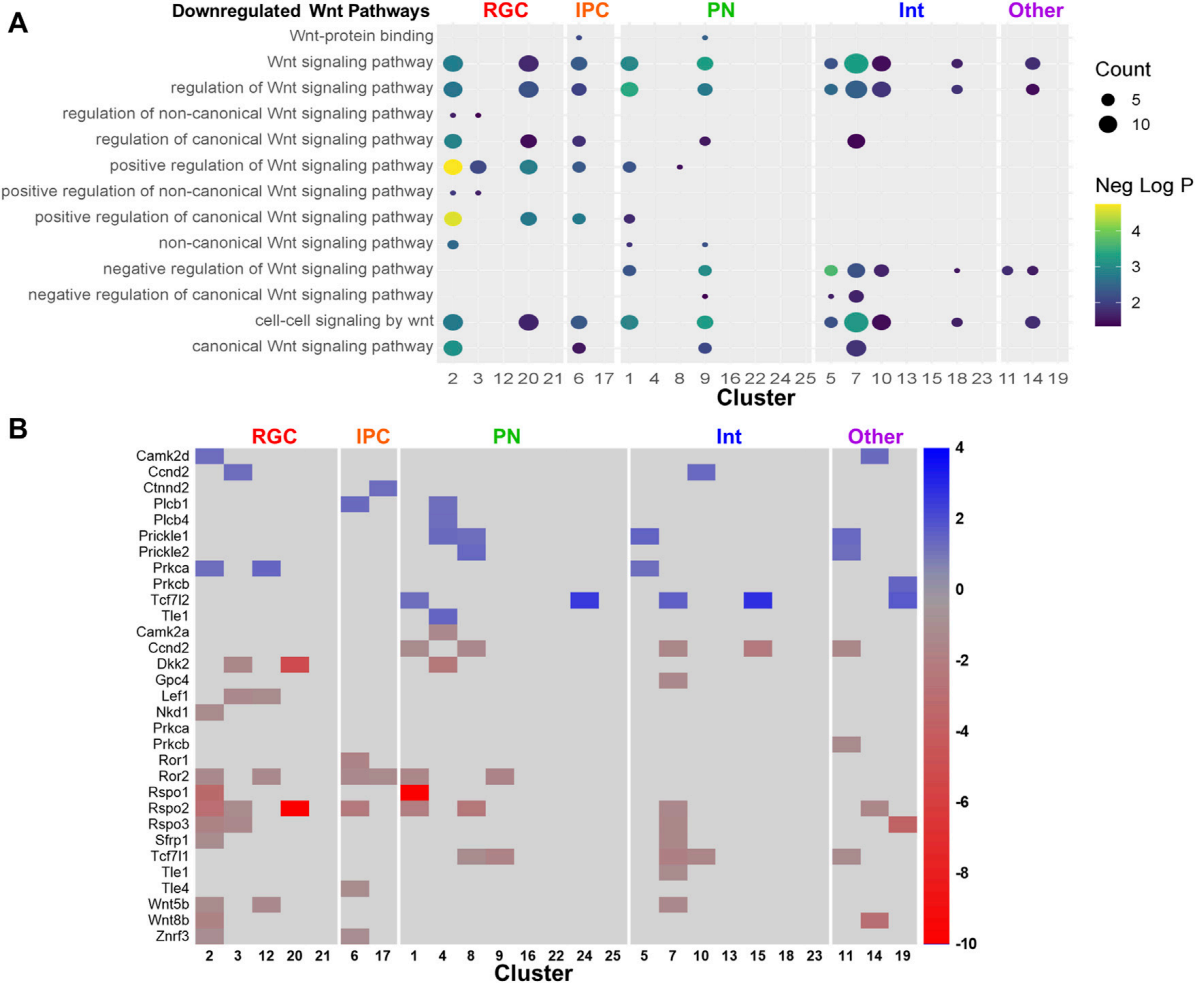
Wnt signaling pathways and genes altered by PAE. **(A)** Dot plot downregulated Wnt-related pathways. **(B)** Heatmap of the fold-change of Wnt signaling related genes.

Pathway analysis was performed on the downregulated and upregulated DEGs. Several pathways were recurrently implicated across cell type clusters. The most recurrent downregulated pathways included ones related to biosynthesis and Wnt signaling (Figure 6A). The most recurrent upregulated pathways included ones related to synaptic activity and neuronal differentiation (Figure 6B). Consistent with the trend towards upregulation of gene expression by PAE, pathways recurrent across several cell type clusters were more likely to be upregulated. Several clusters showed downregulation of Wnt-related pathways (Figure 7A). However, the *Wnt*-expressing cortical hem RGC Cluster 21 itself did not show dysregulation of Wnt pathways, implying that downregulation of Wnt-signaling across multiple cell types may be due to a PAE-induced loss of *Wnt*-expressing cells rather than a change in *Wnt* expression in those cells, further supported by findings that no *Wnt* genes were significantly altered in that cluster. Only a subset of clusters showed downregulation of Wnt-related pathways, including Cluster 20, the *Tcf7l2*+ RGC cluster which showed a lower percentage of cells in mitosis by cell cycle analysis. There was no clear correlation between the expression of Wnt pathway transcription factors or receptors (Supplementary Figure S3) and the other clusters showing a downregulated Wnt pathways. Several genes associated with Wnt signaling were significantly altered (Figure 7B) in several overlapping clusters implicated in the pathway analysis. This included several upregulated genes, which may be due to compensatory mechanisms or mechanisms unrelated to Wnt signaling as several genes are involved in overlapping signaling pathways. Overall, several different analyses of these data point towards a role of Wnt signaling in the effects of moderate PAE on the developing cortex.

## 4 Discussion

The present study investigates the effects of moderate PAE on the embryonic mouse cortex using snRNAseq. We found a significant decrease in the percentage of cells in Cluster 21 cortical hem RGCs, a cluster with enriched expression of *Wnt* genes. Cell cycle analysis revealed a significant decrease of cells in the G2/M phase of the cell cycle in Cluster 20 RGCs, a cluster with elevated expression of a transcription factor downstream in the Wnt signaling pathway, *Tcf7l2*. Differential gene expression and pathway analyses support the disruption of Wnt signaling after PAE, with significant decreases in Wnt-related genes and Wnt-signaling pathways in several clusters. In addition to the observed direct effects on Wnt signaling, we observed an increase in differentiation, as measured by pseudotime analysis, in all IPC and PN clusters as well as select RGC and interneuron clusters. We also observed a bias towards increased as opposed to decreased expression of DEGs in IPCs and PNs, which may be a consequence of or contributing factor towards premature neuronal maturation in glutamatergic neurons and their intermediate progenitors. Overall, these findings reveal cell type-specific effects of moderate PAE and support the use of unbiased approaches such as snRNAseq to discriminate these actions in the developing cortex.

Given the multiple actions of Wnt signaling across all stages of brain development, it is not surprising that a decrease in the number of Wnt-expressing cells may have amplified affects across multiple cell types in the brain. Wnt signaling has been implicated in the effects of PAE in multiple studies, however the direction of effect has varied across different models with some showing a downregulation of Wnt signaling, as we found, (Vangipuram and Lyman, 2012; Tong et al., 2013; Mandal et al., 2015; Subbanna and Basavarajappa, 2020), but some reporting upregulation (Singh et al., 2009; Fischer et al., 2021). These discrepancies are likely due to differences in the model systems used as well as exposure paradigm. We hypothesize that the observed decreased percentage of *Wnt*-expressing cortical hem RGCs resulted in the decreased proliferation of *Tcf72l*-expressing RGCs, downregulation of Wnt signaling in multiple cell types, and increased differentiation of IPCs and PNs. The decrease in Cluster 21 cortical hem RGCs was not correlated with an increase in apoptosis, as measured by the expression of *Casp3* as well as the lack of apoptosis-related pathways detected in the GO analysis. Although apoptosis has been implicated in the effects of PAE in multiple studies, this effect appears to be dependent on the developmental time point of exposure and the differentiation state or cell cycle status of the cell. Neural progenitor cells in general have been shown to be less affected by ethanol-induced cell death (Santillano et al., 2005). Furthermore, previous studies have shown that compared to very high doses of EtOH, more moderate doses, such as that used in this study, do not result in significant cell death (Fischer et al., 2021). As already mentioned, apoptosis may not be accurately detected using sc- and snRNAseq. Therefore, whether or not the decrease in Cluster 21 after PAE was due to apoptosis cannot be readily determined from this data. The decrease in *Wnt*-expressing cortical hem RGCs by ethanol in this study may be due to decreased proliferation, however, cell cycle analysis suggests no changes in the percentage of cells in the G2/M phase, and pathway analysis did not reveal changes in pathways related to proliferation. The effects of ethanol on apoptosis or proliferation in this cell type may have occurred at an earlier time during PAE, and the observed decrease in cells may be a consequence of prior cell death or inhibited proliferation. Because the snRNAseq was performed after prolonged exposure, the acute effects of PAE cannot be readily discerned and findings here are more representative of process related changes. Therefore, the mechanism by which PAE decreased the percentage of cortical hem RGCs may require the examination after acute exposure.

The increased differentiation in select RGC and interneuron cell types and all IPC and PN cell types is consistent with previous studies showing premature neuronal maturation after PAE both during developmental neurogenesis (Camarillo and Miranda, 2008; Kim et al., 2010) and in the mature brain (Gil-Mohapel et al., 2011; Olateju et al., 2018). Differences in the effect of PAE on neuronal differentiation may be due to differences in the markers of differentiation used in each study. By utilizing pseudotime analysis, multiple transcriptional indices of differentiation are accounted for in the generation pseudotime scores, allowing for the quantification of differentiation not limited to the expression of a single or subset of genes. Previous scRNAseq pseudotime analysis in mouse PAE models similarly showed an effect on differentiation, however this affect was sex-dependent and showed both increases and decreases in pseudotime scores across a variety of cell types, with females exhibiting acceleration (Salem et al., 2021). Males and females were combined across treatment groups in this study, and the low number of samples when separating sexes does not allow for that comparison. The mechanism by which PAE accelerated differentiation is unknown, however some speculations include increased differentiation as a compensatory mechanism to the deleterious effects of PAE, alterations in GABA signaling induced by the activation of GABA receptors by ethanol, and the degradation of miRNAs that regulation neuronal maturation (Gil-Mohapel et al., 2011; Almeida et al., 2020).

Wnt signaling can both inhibit or promote the differentiation of embryonic stem cells to neurons, depending on the stage of differentiation, with Wnt activation suppressing neuronal differentiation at earlier stages and enhancing differentiation at later stages (Nordin et al., 2008). Consistent with the inhibitory Wnt effect on differentiation during early development, PAE during neurulation and the onset of neurogenesis in this study resulted in decreased Wnt signaling, presumably caused by a decrease in Wnt expressing cells, which may have consequently led to the increased differentiation of projection neurons observed in this study. This hypothesis requires further investigation wherein Wnt levels would be manipulated. A recent *in vitro* study performed on differentiating neural stem cells reported that PAE induced increases in markers for differentiation that were correlated with an increase in Wnt signaling (Fischer et al., 2021), however this study similarly did not investigate whether this was a causal relationship. The premature neuronal maturation observed here may also be an effect of PAE independent from the effect on Wnt signaling. As ethanol acts through a variety of cellular pathways, the effects of PAE on differentiation may be due to a variety of mechanisms, many of which may be unrelated to Wnt signaling.

Although we observed that interneuron cell types were least affected by PAE, numerous studies have shown that PAE alters interneuron migration and interneuron numbers in the cortex; however, past studies suggest PAE has a larger effect on late-generated GABAergic cells (Isayama et al., 2009). In addition, disrupted interneuron migration in the cortex that has been observed in several studies apparently cannot be readily assessed using transcriptomics. A PAE scRNAseq study by Salem et al. similarly reported lesser effects on interneurons, except for a few clusters. Consistent with that study, we found that PAE affected only a few interneuron clusters. Specifically, Cluster 7 interneurons, enriched for expression of lncRNA *Snhg11*, showed the highest number of both total and upregulated DEGs and was one of two interneuron clusters that showed increased pseudotime scores after PAE. While the function of the lncRNA in the brain is undetermined, its role in oncogenesis involving the activation of Wnt signaling further implicates Wnt-mediated effects of PAE.

Limitations of this study include using i.p. injections as the route of administration, which is more temporally and quantitative precise but less physiological relevant compared to voluntary ethanol feeding paradigms. Additionally, the limited biological replicates restricted stratification of analyses by sex, which was an important confounding variable in a previous scRNAseq study modeling PAE (Salem et al., 2021). While these results provide several lines of evidence for Wnt dysregulation, the mechanism by which ethanol modulates Wnt signaling is unknown and requires further manipulation or reversal studies in this model system. Furthermore, whether the molecular affects observed here have prolonged effects on brain development and ultimately the cognitive function and behavior of the offspring is a future direction of this work.

Because the effects of PAE are dependent on the time, duration, and dosage of exposure, making comparisons across studies varying in any of these parameters challenging. Because pregnant women use alcohol across a wide range of consumption levels for varying durations and at different times of pregnancy, modeling different levels and timeframes of exposure is important for disentangling the multiple mechanisms contributing to FASD. The results of this study are most relevant to the effects of moderate alcohol exposure during early brain development, specifically over the course of neurulation to midway through neurogenesis. As up to 50% of women of childbearing age take part in moderate alcohol consumption (Tan et al., 2015) and early brain development occurs at a time when women may not know they are pregnant, it is critical to understand the effects of alcohol exposure during early embryonic development. The use of a milder PAE model (once daily 2.5 g/kg) is reflected in the lack of apoptosis or widespread effects of ethanol across all cell types. The use of snRNAseq in such model, however, provides the resolution to identify specific cell type vulnerabilities to even moderate PAE, vulnerabilities that might not be otherwise detected when focusing on specific cell types or averaging the effects in bulk tissue. Overall, these findings reveal cell type-specific changes in response to moderate PAE and implicate potential molecular mechanisms by which certain cell types in the developing brain are vulnerable to PAE.

## Data Availability

The data presented in this study are deposited in the GEO repository, accession number GSE211534.
