# Supplementary figures and images for: Cell type-specific changes in Wnt signaling and neuronal differentiation in the developing mouse cortex after prenatal alcohol exposure during neurogenesis
Source: Front Cell Dev Biol. 2022 Dec 5;10:1011974. doi: 10.3389/fcell.2022.1011974 (PMC9761331; doi:10.3389/fcell.2022.1011974)

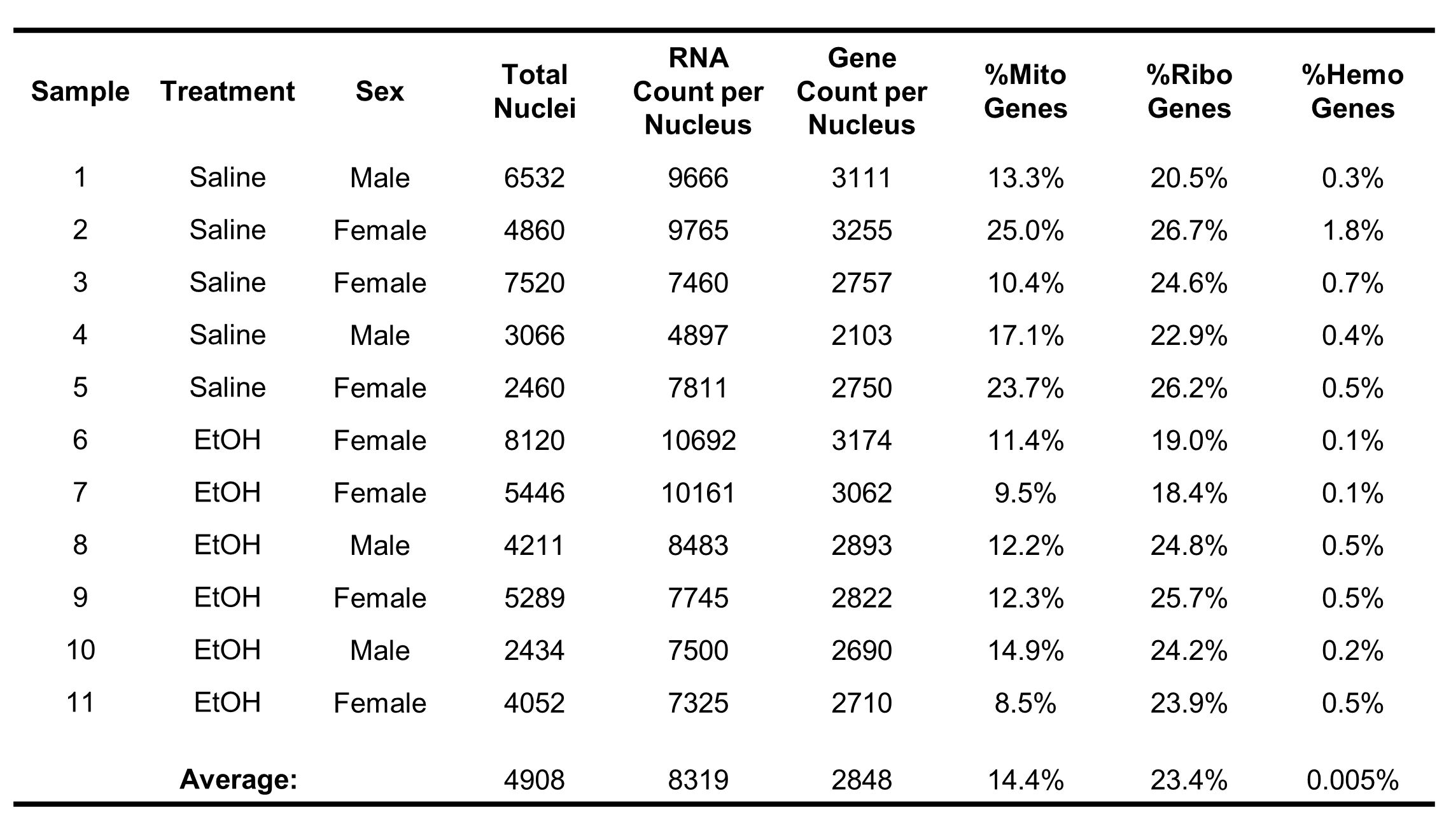

Supplement: Supplementary file 1 [file Image6.tif]

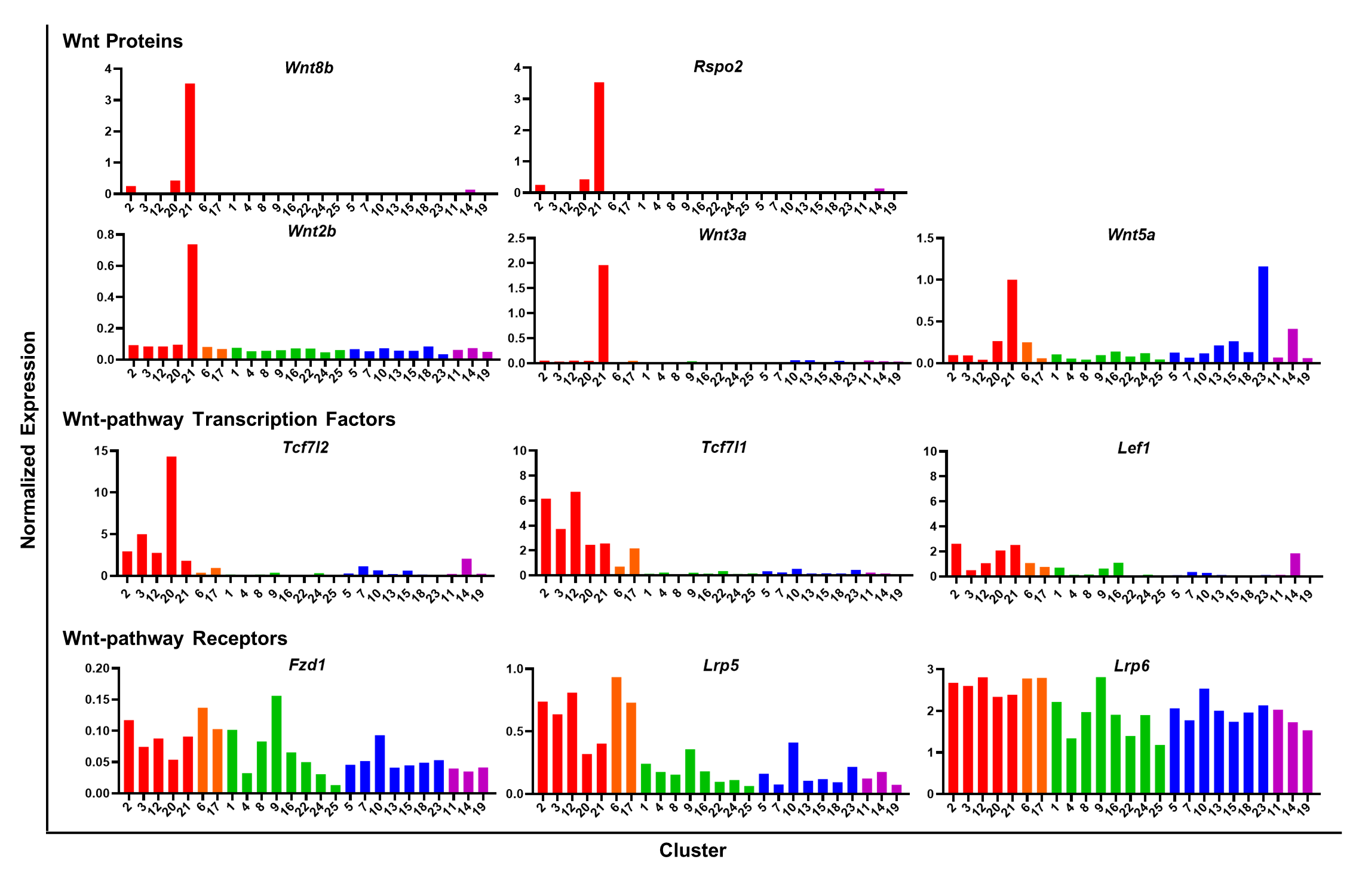

Supplement: Supplementary file 2 [file Image3.tif]

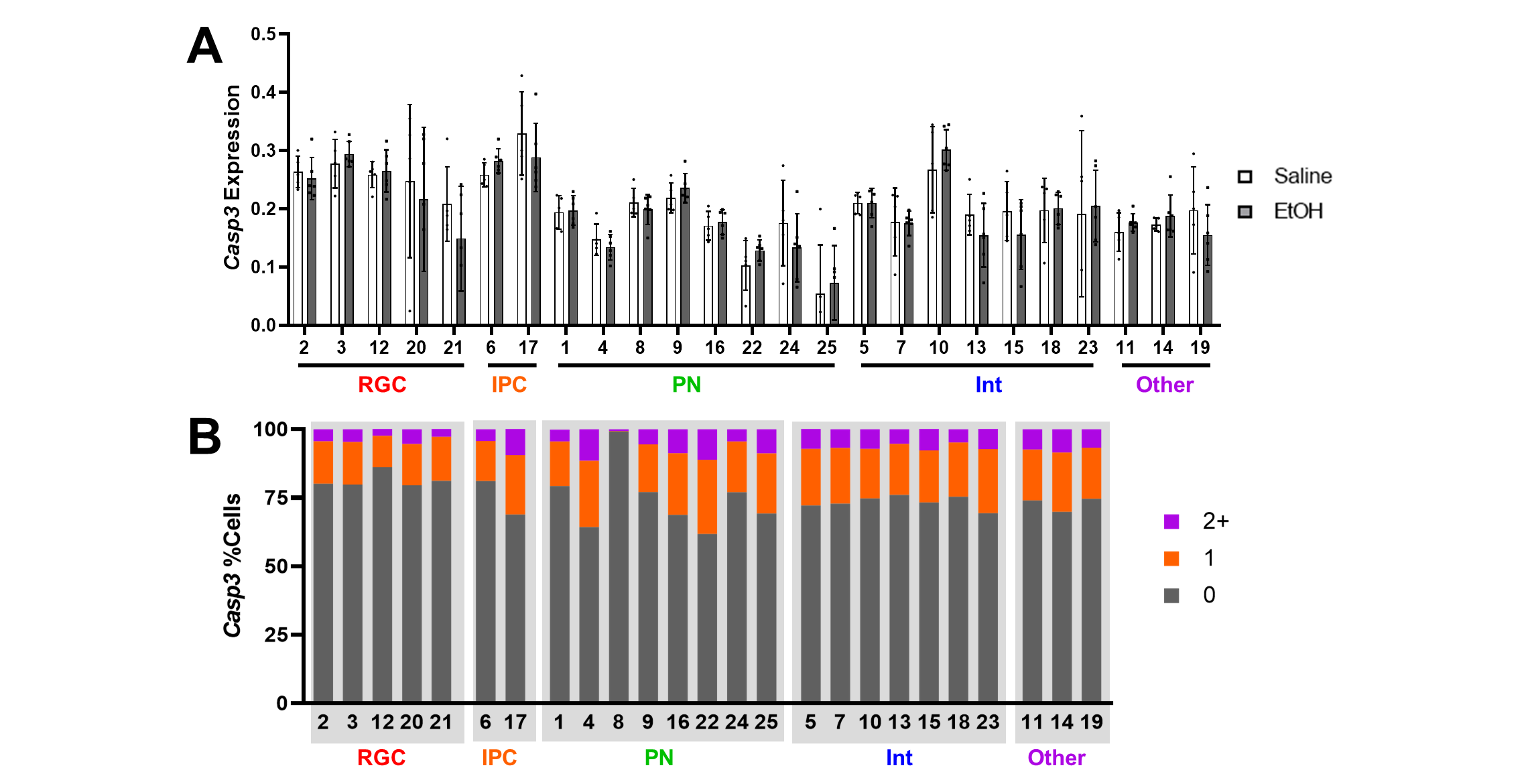

Supplement: Supplementary file 3 [file Image4.tif]

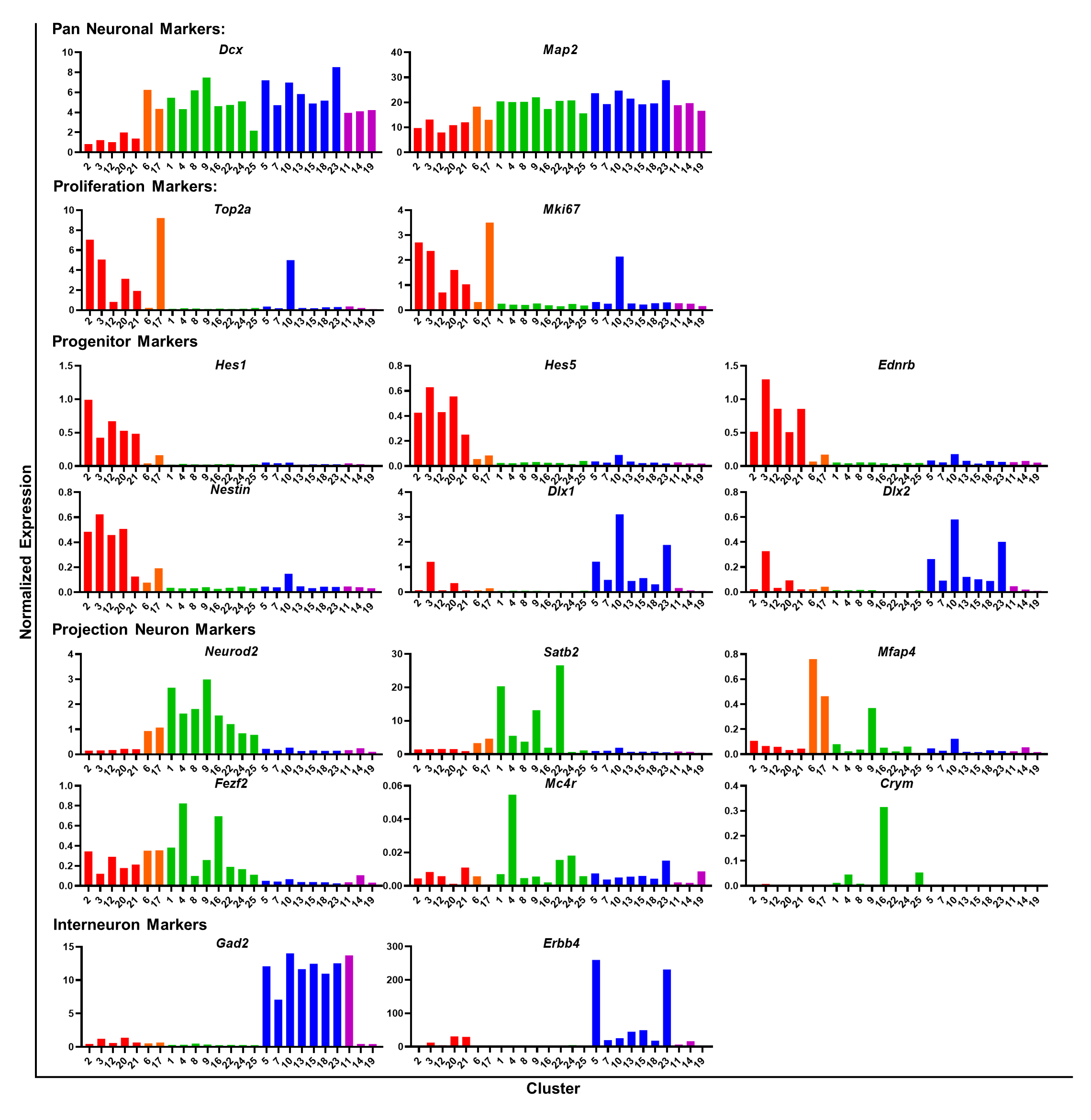

Supplement: Supplementary file 4 [file Image2.tif]

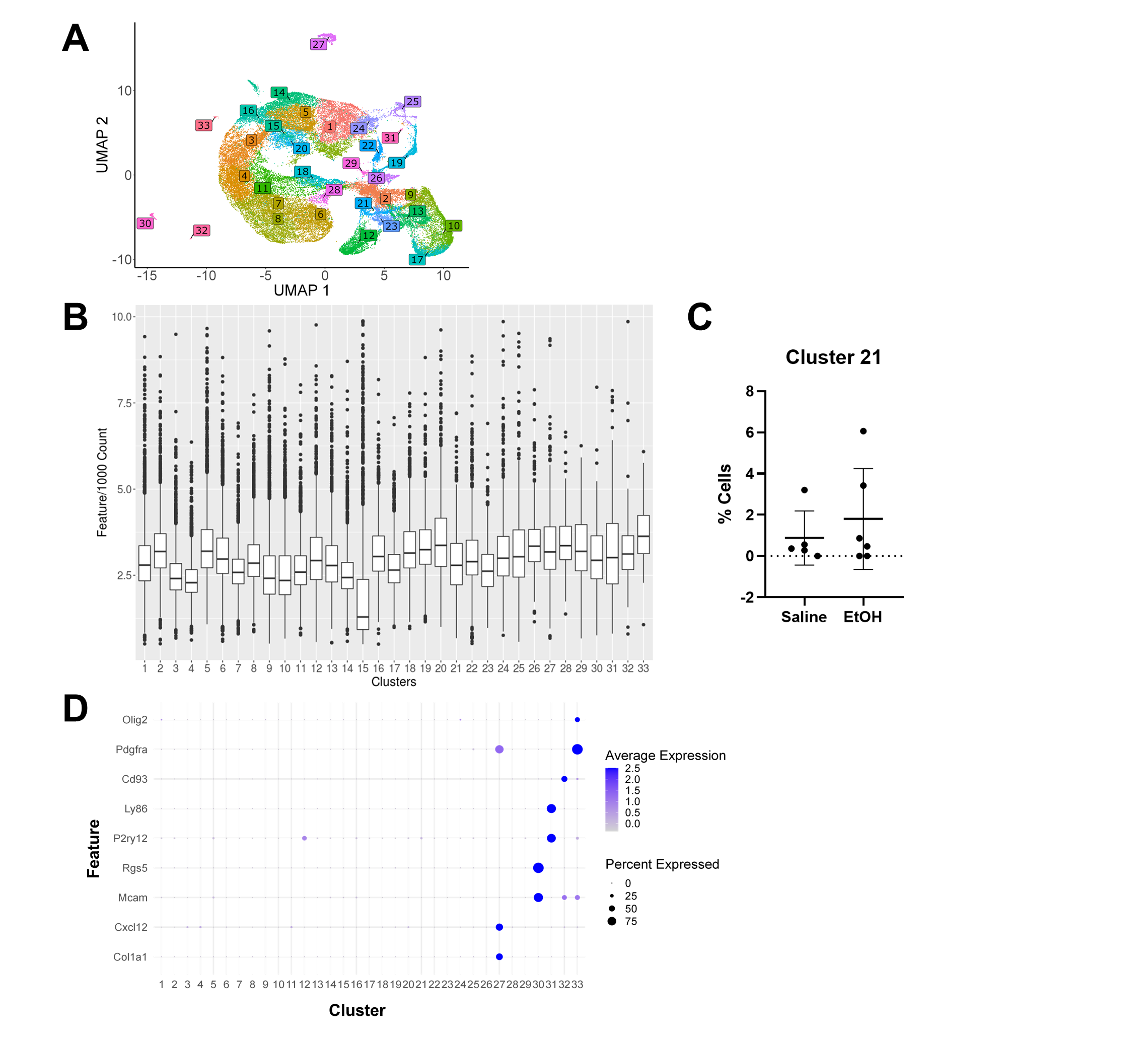

Supplement: Supplementary file 5 [file Image1.tif]

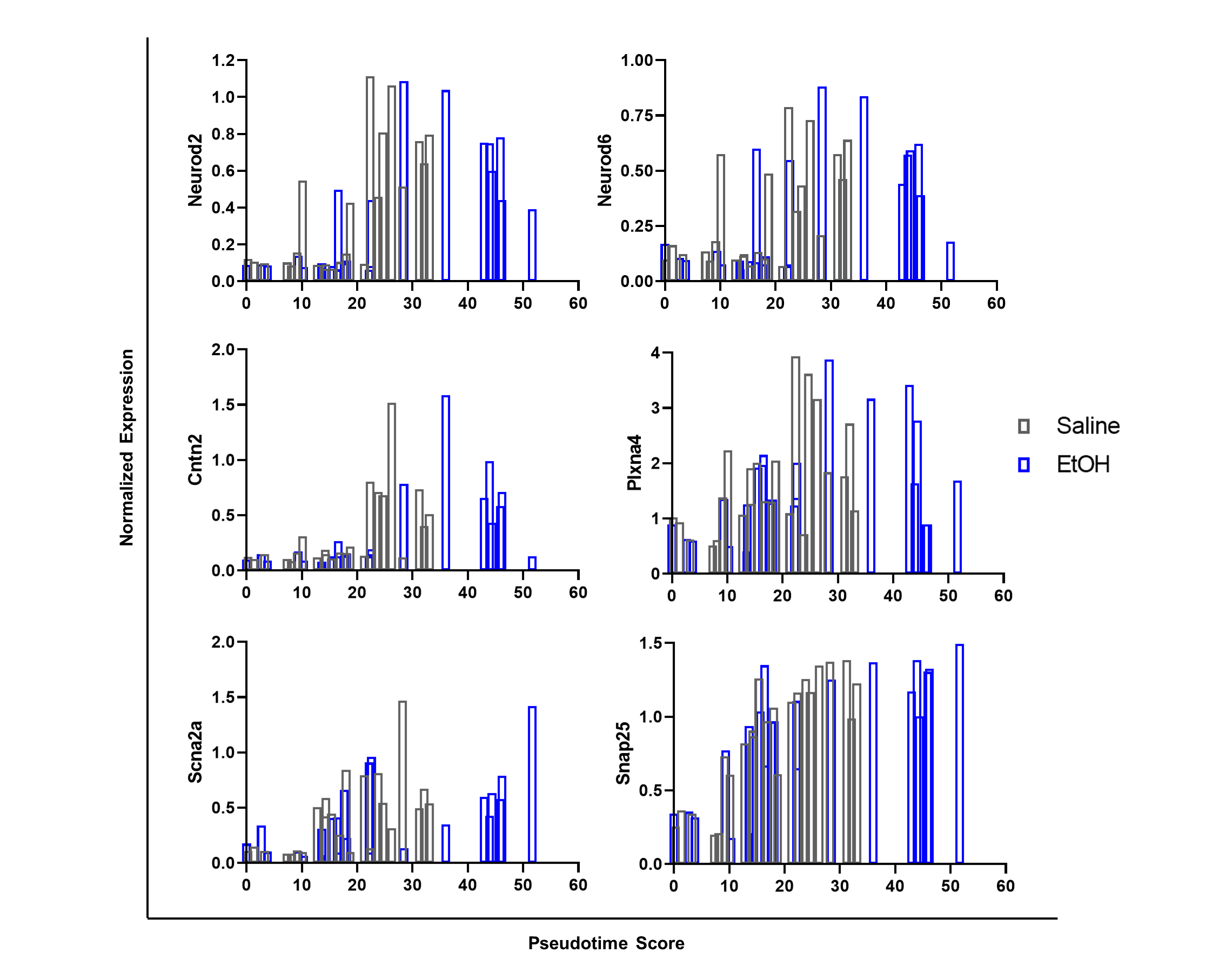

Supplement: Supplementary file 6 [file Image5.tif]
